# Polypyrrole-Grafted Coconut Shell Biological Carbon as a Potential Adsorbent for Methyl *Tert*-Butyl Ether Removal: Characterization and Adsorption Capability

**DOI:** 10.3390/ijerph14020113

**Published:** 2017-01-24

**Authors:** Shanshan Li, Keke Qian, Shan Wang, Kaiqiang Liang, Wei Yan

**Affiliations:** 1 Department of Environmental Science and Engineering, Xi’an Jiaotong University, Xi’an 710049, China; shanshan0320@xjtu.edu.cn (S.L.); 15637390053@163.com (K.Q.); wangshanw@stu.xjtu.edu.cn (S.W.); 2 Research Institute of Yanchang Petroleum (GROUP) Co. Ltd., Xi’an 710075, China; lkq886@163.com

**Keywords:** polypyrrole, active carbon, PPy/GAC, methyl *tert*-butyl ether, carrier, biological filtration

## Abstract

Methyl *tert*-butyl ether (MTBE) has been used as a common gasoline additive worldwide since the late twentieth century, and it has become the most frequently detected groundwater pollutant in many countries. This study aimed to synthesize a novel microbial carrier to improve its adsorptive capacity for MTBE and biofilm formation, compared to the traditional granular activated carbon (GAC). A polypyrrole (PPy)-modified GAC composite (PPy/GAC) was synthesized, and characterized by Fourier transform infrared spectroscopy (FT-IR) and Brunauer-Emmett-Teller (BET) surface area analysis. The adsorption behaviors of MTBE were well described by the pseudo-second-order and Langmuir isotherm models. Furthermore, three biofilm reactors were established with PPy/GAC, PPy, and GAC as the carriers, respectively, and the degradation of MTBE under continuous flow was investigated. Compared to the biofilm reactors with PPy or GAC (which both broke after a period of operation), the PPy/GAC biofilm column produced stable effluents under variable treatment conditions with a long-term effluent MTBE concentration <20 μg/L. *Pseudomonas aeruginosa* and *Acinetobacter pittii* may be the predominant bacteria responsible for MTBE degradation in these biofilm reactors.

## 1. Introduction

Methyl *tert*-butyl ether (MTBE) is used as an effective gasoline oxygenate because of its low production cost, high octane rating capability, and miscibility with other gasoline components. The widespread use of MTBE has resulted in increased reports of groundwater contamination, thus, the development of technology to eliminate MTBE contamination has become a priority [1,2]. Adsorption by granular activated carbon (GAC) is effective for removing MTBE [3,4], however, this treatment option is costly because it requires frequent carbon replacement (the MTBE adsorptive capacity of GAC is only 6 mg/g at an equilibrium concentration of 1 mg/L for Calgon F-400) [5]. MTBE can be biodegraded by acclimated bacteria [6,7,8,9]; however, it is unlikely that biotreatment alone would produce an effluent below the U.S. Environmental Protection Agency’s (EPA) recommended discharge limit of 20 μg/L.

The biological activated carbon (BAC) process removes organic pollutants by a dual-removal mechanism: adsorption on GAC and biological degradation of the adsorbed and dissolved organic pollutants [10]. The dual-removal mechanism of the adsorber enables its long-term operation without periodic GAC replacement [11]. Kim et al. determined that the GAC of the BAC system was capable of maintaining a stable ratio of C/N in the adsorber, which provided an outstanding denitrification performance [12]. Mochidzuki and Takeuchi achieved better biotreatment of plating wastewater in the BAC system because there was less inhibition from the adsorption of some heavy metal constituents [13]. Hu et al. reported the feasibility of the BAC treatment for removing MTBE from groundwater. However, the start-up time was too long, and the amount of MTBE in the stable effluent (50 μg/L) remained above the U.S. EPA’s recommendation for drinking water sources. The high amount of MTBE could be ascribed to the poor attachment of the microbes on the GAC [14].

Polypyrrole (PPy) is a well-known semiconducting polymer that offers high electrical conductivity, environmental stability, and biocompatibility. Hong et al. deposited PPy into the pores of wood-based activated carbon for sulfate removal [15]. Moreover, when PPy is coupled with carrier ions, such as Cl^−^, ClO_4_^−^, NO_3_^−^, and Br^−^, it can exhibit an anion-exchange capacity because of the high mobility of these ions in a polymer matrix. Specifically, when FeCl_3_ is used as the oxidant, its Cl^−^ ions can act as counterions to balance the positive charge on the pyrrole functionalities; the Cl^−^ ion can then be exchanged with anions that appear in the water that is being treated. Zhang et al. showed that PPy has a positive potential below pH 10, which could attach compounds with negative charges, such as the phosphate groups in a bacterial outer membrane [16].

We synthesized an adsorbent by modifying GAC with PPy (PPy/GAC). The objectives of this work were to characterize the adsorbent and investigate its adsorption behaviors for MTBE. A biofilm reactor was established to achieve simultaneous adsorption and biodegradation of MTBE under continuous flow.

## 2. Materials and Methods

### 2.1. Materials

The pyrrole monomer (98%) was purchased from Zhejiang Qingquan Pharmaceutical & Chemical Co., Ltd. (Xianju, China) and distilled under vacuum. The distilled pyrrole was stored at 4 °C under the protection of nitrogen before use. Coconut shell-based GAC (40–80 mesh) was purchased from Sigma (Sigma-Aldrich Corp., St. Louis, MO, USA). Prior to use, the GAC was washed several times with deionized water to remove carbon fines, dried in an oven at 105 °C, and sterilized by autoclaving. MTBE was obtained from Sinopharm Chemical Reagent Co., Ltd. (Shanghai, China). Microbial consortium ERS, cultured in mineral salt medium (MSM) was used to inoculate the different adsorbent columns [17].

### 2.2. Preparation of PPy and PPy/GAC Composites

The PPy particles were synthesized in citric acid solution with FeCl_3_ as an oxidant using the soft-template method [18]. The typical synthetic process was performed as follows. First, citric acid monohydrate (13.44 g) was dissolved in deionized water (400 mL) in a three-necked flask equipped with a mechanical stirring device and stirred for 30 min at room temperature. Then, pyrrole monomer (1.2 mL) was added to the suspension. The FeCl_3_ solution (3.0 M, 20 mL) was slowly added to the mixture in a dropwise manner with stirring (30 min). The polymerization process continued for another 24 h. Finally, the resulting black precipitate was washed several times with deionized water until the filtrate became colorless and dried in a vacuum oven at 50 °C for 24 h.

The PPy/GAC composites were synthesized by the chemical oxidative polymerization of the pyrrole monomer (different concentrations) in the GAC suspension solutions with FeCl_3_ as the oxidant. First, the pre-treated GAC (2.5 g) was added to a pyrrole monomer solution (25 mL) at different concentrations (0.1, 0.2, 0.5, and 1 M), with magnetic stirring for 16 h. After filtration, a FeCl_3_ solution (50 mL, 2.0 M) was added dropwise to the formed suspension, and the mixed solution was stirred for another 6 h. Finally, the PPy/GAC composites were filtered and washed with deionized water. The prepared composites were dried at 50 °C for 24 h. The synthesized composites were named 0.1-PPy/GAC, 0.2-PPy/GAC, 0.5-PPy/GAC, and 1-PPy/GAC according to the concentration of the pyrrole monomer solution that was used during the preparation.

### 2.3. Characterization

Fourier transform infrared (FT-IR) spectra of the samples were measured using the KBr pellet method with a TENSOR 37 FT-IR spectrophotometer (Bruker, Billerica, MA, USA) in the range of 4000–400 cm^−1^. The sample morphology was characterized by scanning electron microscopy (SEM, JSM-6700F, JEOL Ltd., Akishima, Japan). The Brunauer-Emmett-Teller (BET) surface area (*S*_BET_), total pore volume (*V*), and average pore radius (*R*) were measured at 77 K using a nitrogen adsorption apparatus (Builder SSA-4200, Beijing, China).

### 2.4. Adsorption Experiments

All adsorption experiments were performed in 50-mL serum bottles sealed with polytetrafluoroethylene (PTFE) stoppers at 30 °C. The suspension containing the 20 mL MTBE solution (1 mg/L) and 2 g/L of the adsorbent was stirred for 2 h then centrifuged at 4000 rpm for 5 min. The MTBE concentration in the supernatant was determined according to the following method. The effect of the dose of adsorbent on the MTBE adsorption was studied by adding different masses (0.5–10 g/L) of the adsorbent to a 1 mg/L MTBE solution. The adsorption rate, *R* (%), and the amount of dye molecules adsorbed on the adsorbents, *Q_t_* (mg/g), at certain times, *t*, were calculated from the following equations:

(1) $$R\;\left(\%\right)=\frac{C_{0}-C_{t}}{C_{0}}\times 100$$

(2) $$Q_{t\;}\left(\mathrm{mg}/g\right)=\frac{C_{0}-C_{t}}{M\times V}$$

where *C*_0_ is the initial concentration of MTBE (mg/L), *C_t_* is the residual concentration at the special time point, *t* (mg/L), *V* is the solution volume (L), and *M* is the adsorbent mass (g).

The adsorption equilibrium of MTBE (1 mg/L) was evaluated at 25 °C. The adsorption kinetics was investigated using pseudo-first-order and pseudo-second-order models, according to the following equations, respectively:

(3) $$\mathrm{lg}\left(Q_{eq}-Q_{t}\right)=\mathrm{lg}Q_{eq}-\frac{K_{1}}{2.303}t$$

(4) $$\frac{t}{Q_{t}}=\frac{1}{K_{2}Q_{eq}^{2}}+\frac{t}{Q_{eq}}$$

where *t* is the adsorption time (min), *K*_1_ (min^−1^) and *K*_2_ (g/mg/min^0.5^) are the rate constants for the pseudo-first-order and pseudo-second-order models, respectively, and *Q_eq_* (mg/g) is the adsorption amount at the equilibrium state.

Adsorption isotherms for MTBE on different adsorbents at 25 °C were obtained by mixing different concentrations of MTBE solutions (0.1–50 mg/L) with 2 g/L of the adsorbent. The Langmuir and Freundlich isotherms are described in linear forms according to Equations (5) and (6), respectively:

(5) $$\frac{C_{eq}}{Q_{eq}}\;\left(g/L\right)=\frac{1}{Q_{max}K_{L}}+\frac{C_{eq}}{Q_{max}}$$

(6) $$\log Q_{eq}\;\left(\mathrm{mg}/g\right)=\log K_{F}+n\log C_{eq}$$

where *C_eq_* (mg/L) is the MTBE equilibrium concentration, *Q_max_* (mg/g) is the maximum adsorption capacity, *K_L_* (L/mg) is a constant that is related to the heat of adsorption, *K_F_* (mg/g) represents the adsorption capacity when *C_eq_* equals 1, and *n* represents the dependence of the degree of adsorption on the equilibrium concentration.

### 2.5. Laboratory Column Experiments

**Figure 1:**
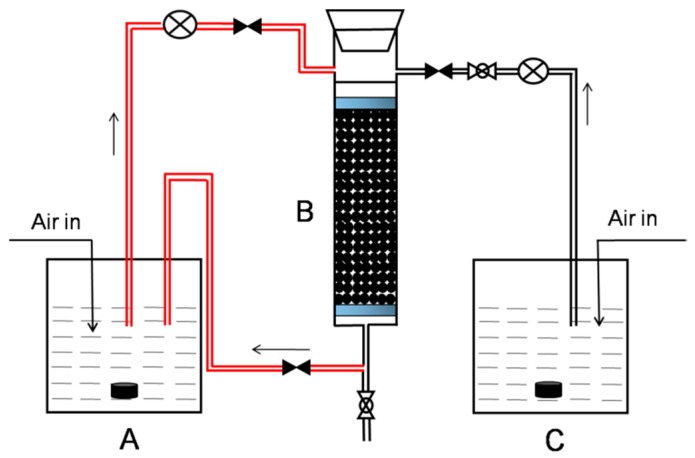
Schematic diagram of the laboratory column experiments showing the culture vessel (**A**); reactor with different adsorbents (**B**); and MTBE-containing (Methyl *tert*-butyl ether) solution vessel (**C**). The red line represents recirculation through the feeding vessel (closed mode operation), and the black line represents circulation through the column only (open mode operation). Both the vessel and reactor were maintained at 30 °C.

Different adsorbents were packed into separate Plexiglass columns (length = 25 cm, internal diameter = 2.0 cm) at a bed height of 15 cm. A schematic of the column setup is presented in Figure 1, and the conditions of the column experiments are listed in Table 1. The reactor was maintained at 30 °C. Each column was inoculated with 500 mL of the MTBE-grown consortium ERS, introduced using a peristaltic pump from an aerated feeding vessel (A). The reactor was operated initially in recycle mode (closed system) for three days to allow the inoculum to attach to the adsorbents. The decrease (>90%) in optical density (OD_600_) of the circulating inoculum was used as an indication of biomass loading onto the adsorbent column. At three intervals over the four-week period, the spent medium was removed from the reactor and replaced with identical fresh medium supplemented with MTBE (1 mg/L) with an empty bed contact time (EBCT) of 28.5 min. In open mode operation, each column was continuously fed by fresh MSM (mineral salts medium) with MTBE at a concentration of 1 mg/L according to the conditions in Table 1. Water samples were taken from the influent and effluent of each column at regular intervals to analyze the MTBE concentration.

### 2.6. Biofilm Microbial Community Analysis

The bacterial communities attached to the different adsorbent columns were characterized by 16S-rRNA-based sequence analysis. Bacteria were recovered from the different adsorbents and were purified by repeated subculturing on nutrient agar and minimal salts agar supplemented with MTBE. Genomic DNA from a single strain was extracted using the Ezup Column Bacteria Genomic DNA Purification Kit (Sangon Biotech, Shanghai, China) according to the manufacturer’s instructions. A partial 16S rRNA sequence was amplified from the genomic extraction with universal primers 27F (5′-AGAGTTTGATCCTGGCTCAG-3′) and 1492R (5′-GGTTACCTTGTTACGACTT-3′). The amplification reaction mixtures (50 µL) contained 1 µL of the template, 1.5 U of PrimeSTAR HS DNA Polymerase (Takara Biotech, Dalian, China), 10 µL of 5× PrimeSTAR buffer, 2 µL each of forward and reverse primers (10 µM), and 5 µL of a dNTP mixture (2.5 mM). Amplifications were performed using a T100 Thermal Cycler (Bio-Rad Laboratories, Hercules, CA, USA) at the following conditions: 98 °C for 5 min, 30 cycles at 98 °C for 10 s, 55 °C for 5 s, 72 °C for 90 s, and a final extension at 72 °C for 7 min. After purification, the polymerase chain reaction (PCR) product was Sanger-sequenced by Sangon Biotech (Shanghai, China).

The DNA sequence was used to identify homologous sequences in GenBank with BLAST. Phylogenetic analysis of the 16S rRNA sequence was performed using the MEGA 5.1 software [19]. Multiple alignments with sequences from GenBank were performed using the Clustal method [20]. A phylogenetic tree was constructed using the neighbor-joining method. The 16S rRNA sequences were deposited in the National Center for Biotechnology Information (NCBI) GenBank database under accession number KT229734-KT229749.

### 2.7. Analytical Methods

MTBE concentrations were analyzed by headspace solid-phase dynamic extraction-gas chromatography-mass spectrometry (HS-SPDE-GC/MS). The liquid culture was collected and centrifuged at 6000 rpm for 10 min. Next, 2 mL of the supernatant was added to a 10-mL glass vial sealed with a screw cap and silicone-polyperfluoroethylene gaskets and immediately underwent HS-SPDE analysis on a CTC CombiPAL-*xt* autosampler (Chromtech, Idstein, Germany) equipped with a polar, polyethylene glycol-coated WAX syringe, as described by Li et al. [17]. The sample that was absorbed on the SPDE syringe was automatically injected into a TRACE GC Ultra (Thermo/Finnigan, Milan, Italy) gas chromatograph equipped with a HP-5ms capillary column (30 m length, 0.25 mm ID, 0.25-μm film; Agilent, Santa Clara, CA, USA) and a TraceISQ (Thermo/Finnigan, Milan, Italy) mass spectrometric detector. The injection port was set in splitless mode and maintained at 260 °C. The oven temperature was maintained at 40 °C for 2 min and then ramped to 120 °C at 5 °C/min. The flow rate of the carrier gas (He, 99.999%) was 1.0 mL/min. The interface and ion source temperatures were maintained at 280 °C and 230 °C, respectively. The mass spectrometer was operated in electron impact mode at 70 eV in selected ion monitoring (SIM) mode at 73 m/z for MTBE. The amount of MTBE was quantified using standards of known concentrations.

All computer-based statistical analyses were performed with the SPSS 16.0 software for Windows (SPSS Inc., Chicago, IL, USA). A one-way analysis of variance (one-way ANOVA) was used to assess the differences between treatments at a significance level of 0.01 or 0.05.

## 3. Results and Discussion

### 3.1. Characterization of the Adsorbents

**Figure 2:**
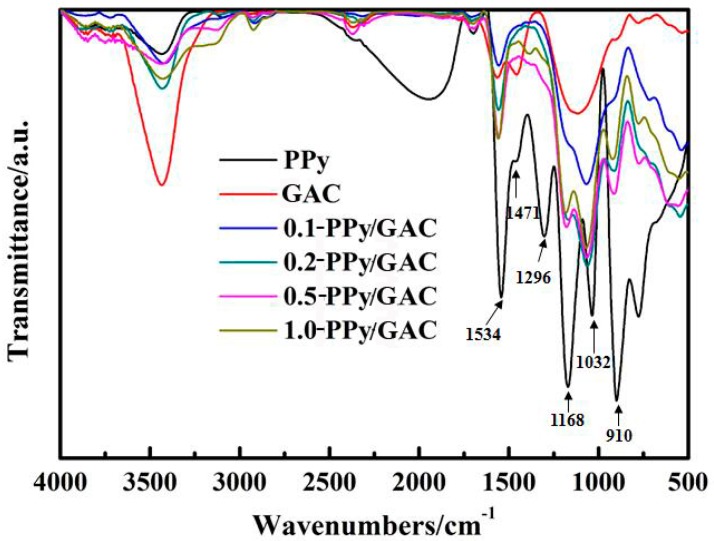
FT-IR spectra of the prepared adsorbents.

The FT-IR spectra (Figure 2) show the C=C ring stretching band of pyrrole at 1534 cm^−1^ (quinonoid structure of PPy). The band at 1471 cm^−1^ is attributed to the C-N stretching vibration in the pyrrole ring. Additionally, the peaks at 1296, 1168, and 1032 cm^−1^ are ascribed to the in-plane -C-H vibrations, and the peak at 910 cm^−1^ is for the =C-H out-of-plane vibration [18,21]. This indicated that the backbone of PPy/GAC is similar to that of PPy prepared by the conventional route, and PPy was successfully sorbed throughout the pore matrix of the GAC [18].

### 3.2. Adsorption Properties of the Adsorbents

The specific surface area of the adsorbents, which is related to the adsorption properties, was analyzed. The BET surface area (*S*_BET_), total pore volume (*V*), and average pore radius (*R*) of the different adsorbents varied with respect to the pyrrole monomer concentration (Table 2). The BET surface area of PPy (*S*_BET_ = 58.67 m^2^/g) was extremely lower than that of the traditional GAC. Furthermore, both the BET surface area and the pore volume decreased with increasing pyrrole monomer concentration. These pore volume decreases confirmed that PPy successfully sorbed throughout the pore matrix of GAC; therefore, the FeCl_3_ catalyst likewise caused polymerization throughout this pore matrix. The decreased pyrrole monomer concentration enhanced the BET surface area, and the pore volume benefitted from the adsorption of MTBE. However, the reduced pore radius might have weakened the adsorption capacity of MTBE and the microbes.

**Figure 3:**
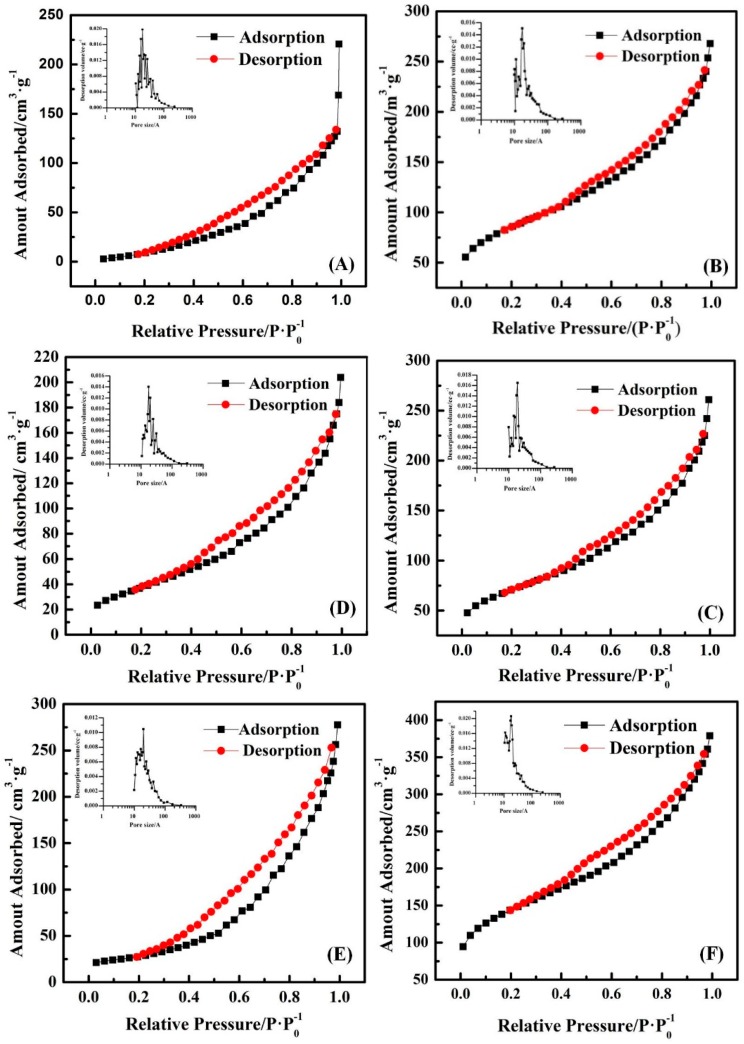
Adsorption-desorption isotherms of N_2_ of the adsorbents. PPy (**A**); 0.1-PPy/GAC (**B**); 0.2-PPy/GAC (**C**); 0.5-PPy/GAC (**D**); 1-PPy/GAC (**E**) and GAC (**F**).

The micropores (<20 Å) mostly consisted of the pore volume of GAC. Figure 3 shows that GAC possessed well-developed micropores. Micropores were also well developed for 0.1-PPy/GAC and 0.2-PPy/GAC, but the mesopores (20–500 Å) were less developed. However, mesopores were well developed for 0.5-PPy/GAC, 1-PPy/GAC, and PPy. The different pore size distributions in the adsorbents might be caused by deposits of the pyrrole monomer.

**Figure 4:**
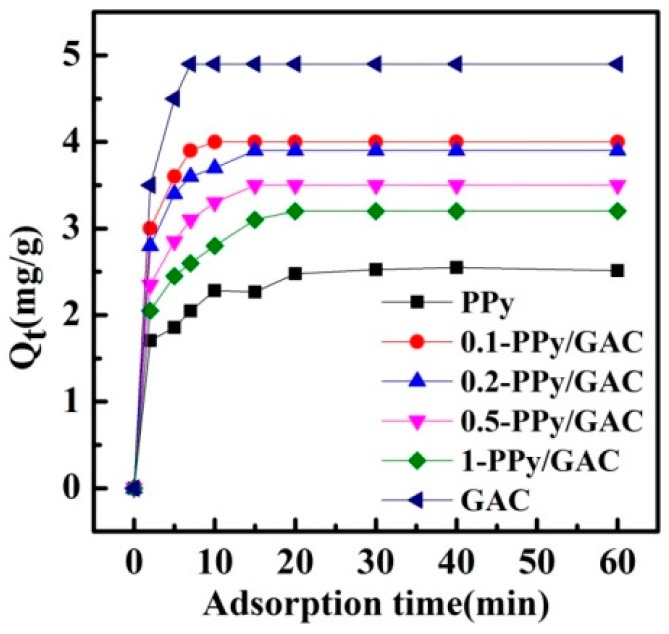
Adsorption equilibrium curves of MTBE on different adsorbents.

Apart from possessing a high adsorption capacity, it is essential for an adsorbent to exhibit rapid adsorption kinetics for removing the adsorbate from the solution. Figure 4 shows the influence of the contact time on the adsorption of MTBE for the six adsorbents. The adsorption of MTBE on GAC was relatively rapid, and PPy required a longer adsorption equilibrium time than the PPy/GAC composites. Additionally, the adsorption capacity decreased with increasing pyrrole monomer concentration in the synthesis. The increase of the pyrrole monomer will fill the available pores of the GAC, but decrease the BET surface area.

Moreover, the adsorption kinetics of MTBE was fitted by the pseudo-first-order and pseudo-second-order models. The related parameters for the adsorption kinetics were calculated and are listed in Table 3. According to the values of the correlation coefficients, the adsorption behaviors of the four composites can be well described by the pseudo-second-order model (*R*^2^ = 0.9856–0.9984). Furthermore, the calculated values of *Q_eq_* from the pseudo-second-order model were approximately equal to the experimentally obtained values. These results indicate that the adsorption kinetics of the synthesized adsorbents corresponded to the pseudo-second-order model.

The adsorption isotherm was examined to determine the interaction between the adsorbate and adsorbent. The experimental parameters of the Freundlich and Langmuir isotherm models are listed in Table 4. The adsorption behaviors of the MTBE adsorbents are more consistent with the Langmuir isotherm (the correlation coefficient, *R*^2^ is greater for the Langmuir model than that for the Freundlich model). The dimensionless separation factor, *R_L_*, which is an essential characteristic of the Langmuir model for reflecting the favorability of an adsorption process, is used:

(7) $$R_{L}=\frac{1}{1+K_{L}C_{m}}$$

where *C_m_* is the maximum initial concentration of MTBE in the solution. The *R_L_* values were calculated and are listed in Table 4. The values of *R_L_* for the four composites were in the range of 0–0.5, indicating that the adsorption processes were favorable.

Based on the BET analysis, pyrrole monomer concentration, and MTBE-adsorption capacity of the four composites, 0.2-PPy/GAC was chosen for further analysis of the biofilm reactor.

### 3.3. Adsorption and Biodegradation of MTBE in Continuous Flow by the Biofilm Reactor

The microbial consortium ERS possesses a capacity for directly degrading MTBE [6]. In this study, the consortium ERS was used to inoculate three separate biofilm reactors with PPy, 0.2-PPy/GAC, and GAC as the adsorbents, respectively. Initially, the reactor was operated as a closed recirculating system (Figure 1) to establish and maintain the biofilm on the column. Biofilm development occurred on the adsorbent particles under these conditions.

**Figure 5:**
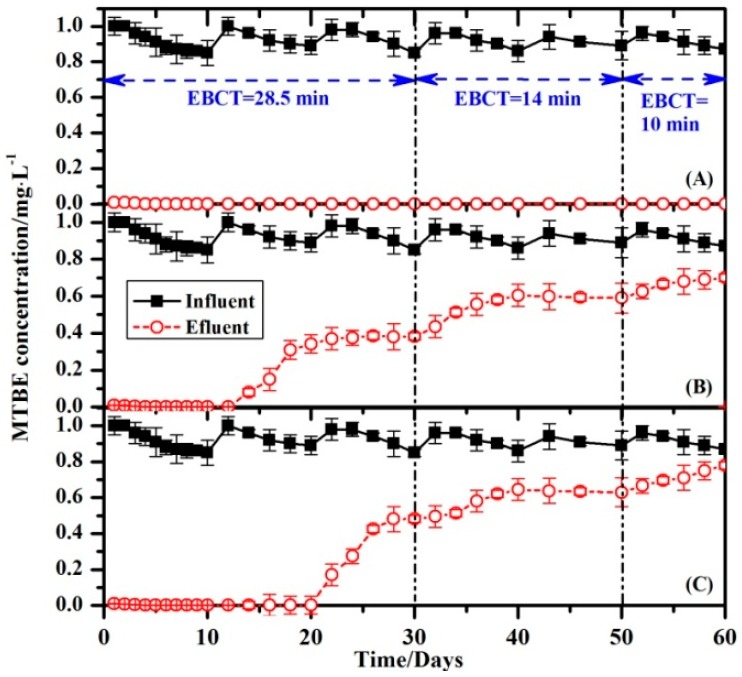
Biological filtration of MTBE through laboratory columns with 0.2-Ppy/GAC (**A**); PPy (**B**); and GAC (**C**) as adsorbents. The error bars represent the standard deviations from replicate measurements.

Adsorption and biodegradation of organic compounds are factors that influence the performance of a column bioreactor. The reactor was operated as an open system (with no recirculation to vessel A) to quantify the degradation of MTBE by the column biofilm in continuous mode (Figure 1). The results for biological filtration of the MTBE through each of the columns are shown in Figure 5.

**Figure 6:**
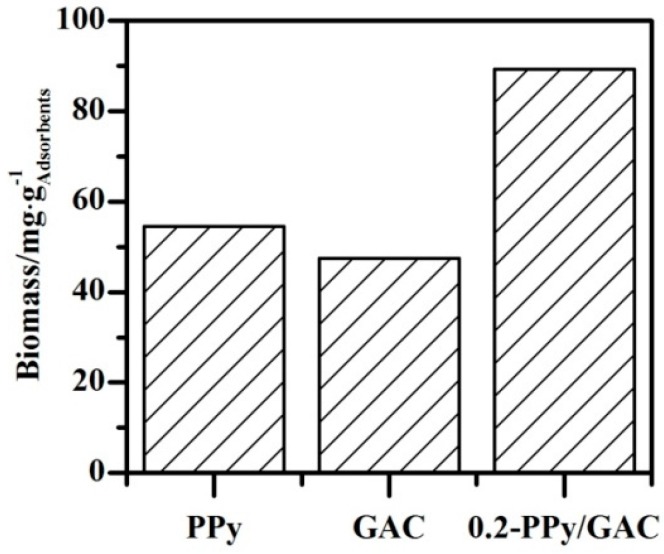
Biomass concentration of three separate columns with different adsorbents.

In this type of system (i.e., the composite of a biofilm and adsorbents), both the adsorption of target compounds to the adsorbents and biotreatment occur simultaneously. In this study, the MTBE that adsorbed to the adsorbents in the column slowly became bioavailable and was mineralized by the biofilm. A major advantage of MTBE adsorption in the column reactor was holding the compound for longer than the hydraulic retention time (HRT), allowing time for the biofilm consortium to effectively mineralize the compound. No MTBE was detected in the effluent of column 0.2-PPy/GAC at any time, even when the operation employed a low EBCT of 10 min (Figure 5). The MTBE removal in column 0.2-PPy/GAC was accomplished primarily by biodegradation of the biofilm because the adsorptive capacity of the 0.2-PPy/GAC adsorbents in the column was only a fraction of the observed MTBE removal. Additionally, the MTBE concentration of the stable effluent at the end of the experiment was <10 g/L, which is below the U.S. EPA’s recommended limit for drinking water. As shown in Figure 6, higher biomass abundances were detected in the 0.2-PPy/GAC column than in the PPy and GAC columns. Then it is probable that a higher concentration of MTBE degrading organisms would have been present in the 0.2-PPy/GAC column. As a result, higher concentration of MTBE-degrading enzyme would be produced, which would increase the MTBE-degrading rate and then improve the removal efficiency in the continuous flow. When the biodegradation of the pre-adsorbed MTBE by the biofilm and the adsorption of MTBE in the matrix that had been released by the degraded MTBE formed a dynamic equilibrium, the biofilm column will exhibited a stable removal efficiency of MTBE. In other words, the biodegradation of pre-adsorbed compounds is dependent on the adsorption and desorption characteristics of the target compounds in the matrix. Carvalho et al. investigated the degradation of 4-chlorophenol (4-CP) by a consortium attached to GAC in a biofilm reactor [22]. They demonstrated that desorption of the adsorbed compounds preceded the biodegradation because the biodegraded fraction never exceeded the fraction that was leachable at the same conditions.

For the reaction of biofilm column B with PPy, no MTBE was detected in the effluent during the first 12 days. However, after 12 days, a time breakthrough was observed in the effluent for the remainder of the experiment. The treatment performance was reduced with an increase of the feed rate using an EBCT of about 20 min (days 30–50). The EBCT was continuously decreased to 10 min, and the MTBE concentration in the effluent increased (Figure 5B). Similar results were observed for the biofilm column of GAC. The results suggest that the MTBE quantity in the influent was larger than the quantity of MTBE degraded by the biofilm on the adsorbents; the residual MTBE that could not be adsorbed or degraded escaped from the column and increased the MTBE concentration in the effluent (Figure 5B,C). Furthermore, the biomass analysis also confirmed this deduction. Lower biomass abundances were detected in the PPy and GAC columns than in the 0.2-PPy/GAC column (Figure 6). Thus, it is probable that a lower concentration of MTBE-degrading organisms would have been present in the PPy and GAC columns.

Lee et al. reported that phenol and chlorinated phenols are more effectively biodegraded by immobilized bacteria than suspended bacteria [23]. The removal of chlorophenol from aqueous solutions treated by microbial cultures was improved when the microbes were immobilized onto compact matrixes. The effect was attributed to the more efficient production of lactase by an established biofilm and the binding of chlorophenols onto the surface of the matrixes, allowing a longer exposure to lactase on the surface and a faster removal rate [24]. Hu et al. attempted to treat MTBE-contaminated water with an effective BAC column; however, the time to establish the biofilm was long, and the treatment capacity was poor [14]. Our studies focus on improving the performance of a biofilm column reactor for MTBE degradation in continuous-flow operation. Continuous biodegradation of MTBE was achieved at an EBCT of 10–30 min. The results demonstrate that the bioaugmentation of acclimated mixed MTBE degraders could be employed to renew and improve the treatment effectiveness of a poor-performing GAC system. They provide an increased stability to the effluent quality and overall system efficiency using a combination of adsorption and biodegradation mechanisms.

### 3.4. Analysis of the Biofilm Community

**Figure 7:**
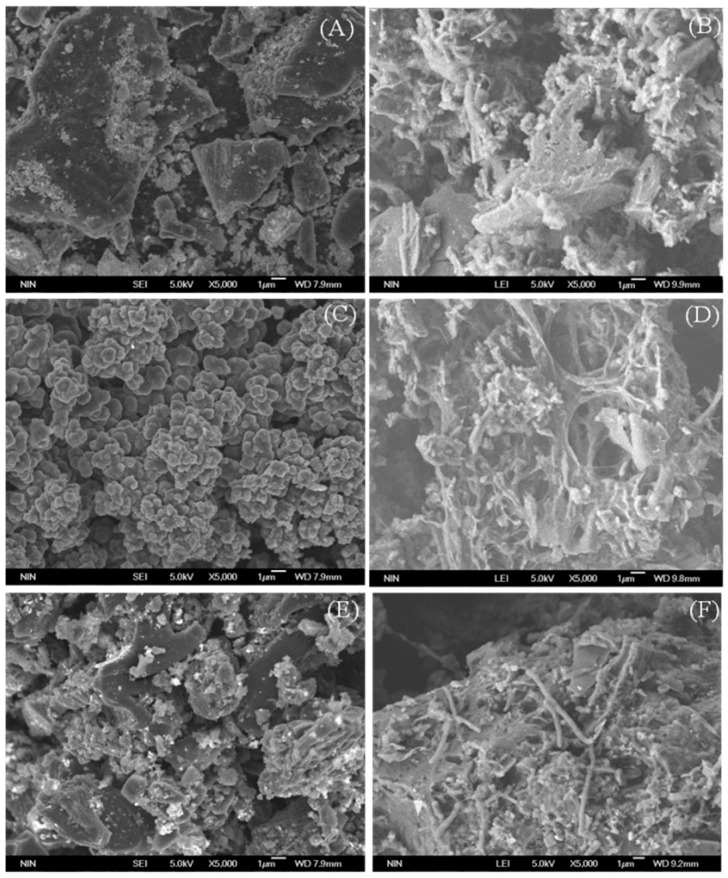
SEM graphs of different adsorbents before (**A**,**C**,**E**) and after (**B**,**D**,**F**) the bioreactor operation. (**A**,**B**) are of GAC adsorbents; (**C**,**D**) are of PPy adsorbents; and (**E**,**F**) are of 0.2-PPy/GAC adsorbents.

No other studies have reported the treatment of MTBE-contaminated water with a microbial consortium attached to PPy-modified GAC. Thus, we needed to identify the microbial communities that were attached to the different adsorbents. At the end of the treatment, the development of the biofilm in the different adsorbent columns was monitored by SEM (Figure 7). Microbial colonization on the three adsorbent particles was evident. Furthermore, aggregation of biomass (mainly rod-shaped bacteria) was visible, and the number of bacterial communities attached to 0.2-PPy/GAC was larger than that on the PPy and GAC adsorbents. This result was consistent with the data for the biomass concentration (Figure 6) and could be ascribed to both the electrostatic attraction of PPy to the microbes and the large specific surface area of GAC.

**Figure 8:**
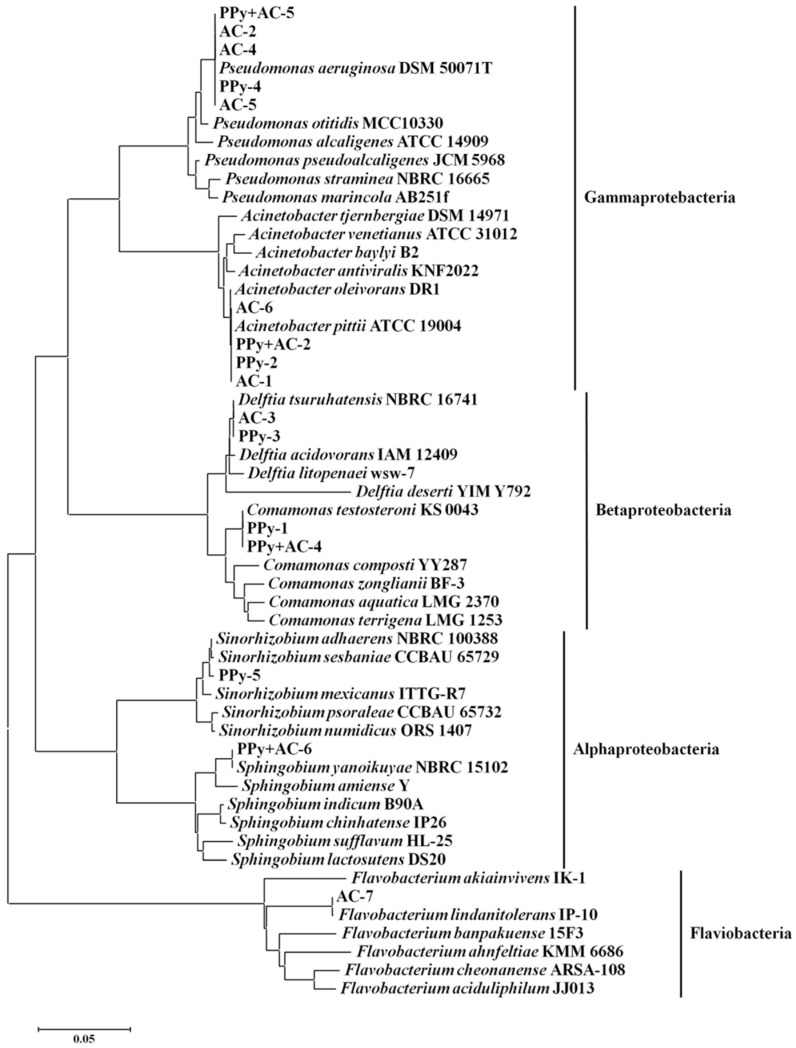
Phylogenetic tree of the bacteria recovered from the different biofilm columns.

Furthermore, a preliminary characterization of the biofilm microbial community was conducted. Several separate single strains were isolated from the biofilms on each column (the 0.2-PPy/GAC, PPy, and GAC columns contained five, four, and four separate single strains, respectively). A preliminary characterization of the recovered bacterial strains showed that all were gram-negative rods. The isolates were identified by 16S-rRNA-based phylogenetic analysis (Figure 8); different biofilm populations attached onto the diverse adsorbents. *Pseudomonas aeruginosa* and *Acinetobacter pittii* were recovered from all three biofilms; they may be the predominant bacteria responsible for MTBE degradation in the biofilm reactors. *Pseudomonas aeruginosa* BM-B-450, which was isolated from an *n*-pentane-adapted consortium, can degrade MTBE [25]. Members of the *Pseudomonas* genus have found innumerous environmental niches on Earth and have demonstrated the ability to mineralize various pollutants, including polycyclic aromatic hydrocarbons (PAHs) [26,27], benzene [28], and alcohols [29,30]. Strains from the *Pseudomonas* genus meet the requirements for industrial biotechnology, including high biomass production and low nutritional demands. Furthermore, numerous typical strains from the genus *Pseudomonas* have been whole-genome sequenced, including *P. aeruginosa* PAO1 [31], *P. putida* KT2440 [32], and *P. mendocina* NK-01 [33]. The broad substrate scope and easy accessibility of genetic bioinformation facilitate the further utilization of *Pseudomonas* genus strains in biotechnology and bioremediation. *Acinetobacter*, which occupies an important niche in nature, is another dominant genus that is ubiquitously distributed in numerous environments, including soil, sediments, water, and contaminated sites [34,35]. Moreover, *Acinetobacter* strains have the ability to mineralize versatile pollutants, such as various long-chain dicarboxylic acids, aromatic compounds, and hydroxylated aromatic compounds [36,37]. The desirable proprieties of *Acinetobacter* strains make them promising alternatives to *Escherichia coli* (a traditional model organism) for metabolism research. One of these desirable properties is the simplicity of genetic manipulation by homology-directed recombination with linear DNA fragments [38].

The isolate belonging to the *Sphingobium* genus was only recovered from the 0.2-PPy/GAC column, *Flavobacterium* sp. was only isolated from the GAC column, *Comamonas* sp. was isolated from the 0.2-PPy/GAC and PPy columns, and *Delftia* sp. was isolated from the GAC and PPy columns. This suggests that biodegradation may be performed in a cooperative fashion by the biofilm bacteria, which may vary in composition accordingly to fluctuations in the feed. In addition, this may indicate that the typical degrading laboratory species may implant less successfully within treatment populations than naturally established bacterial communities in biofilms.

According to the reported results, the most dominant strains in ERS culture belong to *Pseudomonas* sp. (30.4% of the OTUs (Operational Taxonomic Units)) [17], which is consistent with the results from column biofilm. Otherwise, *Serratia* sp., which accounted for 28.1% of the OTUs in ERS culture, had not been detected in the biofilm from all the three kinds of adsorbents. The decreased biodiversity in the mixed ERS after loaded on the carriers may be ascribed to the poor adhesion of some genera, e.g., *Serratia* sp., on the adsorbents.

## 4. Conclusions

In this study, GAC was successfully grafted with PPy by in-situ chemical oxidative polymerization of different concentrations of pyrrole monomer. The adsorption behaviors of MTBE on the composites were described by the pseudo-second-order and Langmuir isotherm models. The adsorption capacities decreased with the increasing concentrations of pyrrole monomer. In the subsequent biofilm reactor experiments, 0.2-PPy/GAC biofilm column exhibited a better and more enduring effect for the elimination of MTBE in continuous flow than PPy and GAC biofilm columns. Furthermore, *Pseudomonas aeruginosa* and *Acinetobacter pittii*, which were recovered from all three biofilms, may be the predominant bacteria responsible for the observed MTBE degradation.

## Figures and Tables

**Table 1:** Laboratory column conditions.

Column	Adsorbents	Time Employed (Days)		
		EBCT 28.5 Min	EBCT 14 Min	EBCT 10 Min
Column A	PPy	0–30	31–50	51–60
Column B	0.2-PPy/GAC	0–30	31–50	51–60
Column C	GAC	0–30	31–50	51–60

EBCT: empty bed contact time; PPy: polypyrrole; GAC: granular activated carbon.

**Table 2:** The textural properties of the adsorbents.

Sample	*S*_BET_ (m^2^/g)	*V* (cm^3^/g)	*R* (nm)
PPy	58.67	0.34	11.7
1-PPy/GAC	96.34	0.33	8.96
0.5-PPy/GAC	141.38	0.32	4.48
0.2-PPy/GAC	255.30	0.41	3.18
0.1-PPy/GAC	307.08	0.42	2.71
GAC	500.88	0.59	2.35

**Table 3:** Parameters for the pseudo-first-order and pseudo-second-order models.

Adsorbent	Pseudo-First-Order Model			Pseudo-Second-Order Model		
	*K*_1_	*Q_eq_*	*R*^2^	*K*_2_	*Q_eq_*	*R*^2^
PPy	0.039	0.217	0.8415	1.42	0.29	0.9935
1-PPy/GAC	0.045	0.379	0.9118	1.21	0.77	0.9856
0.5-PPy/GAC	0.031	2.862	0.8954	0.87	1.31	0.9924
0.2-PPy/GAC	0.027	2.434	0.8223	0.78	3.03	0.9873
0.1-PPy/GAC	0.035	1.458	0.8774	0.67	3.11	0.9961
GAC	0.019	0.978	0.9321	0.34	4.81	0.9984

**Table 4:** Parameters for the Langmuir and Freundlich adsorption isotherm models.

Adsorbent	Langmuir Model				Freundlich Model		
	*Q_max_*	*K_L_*	*R*^2^	*R_L_*	*K_F_*	*n*	*R*^2^
PPy	0.31	0.034	0.9945	0.386	2.163	0.452	0.8583
1-PPy/GAC	0.78	0.075	0.9843	0.211	2.832	0.489	0.9118
0.5-PPy/GAC	1.43	0.099	0.9994	0.168	3.572	0.773	0.8623
0.2-PPy/GAC	2.91	0.177	0.9873	0.102	4.148	1.278	0.8834
0.1-PPy/GAC	3.07	0.192	0.9979	0.094	4.356	1.423	0.9365
GAC	4.74	0.232	0.9990	0.079	4.739	1.547	0.9283
